# Supplementary material for: DUX4 is a common driver of immune evasion and immunotherapy failure in metastatic cancers
Source: bioRxiv. 2023 Jul 11:2023.07.10.548412. Preprint. [Version 1] doi: 10.1101/2023.07.10.548412 (PMC10369889; doi:10.1101/2023.07.10.548412)
Supplement: Supplementary file 1 [file NIHPP2023.07.10.548412v1-supplement-1.pdf]

bioRxiv preprint doi: https://doi.org/10.1101/2023.07.10.548412; this version posted July 11, 2023. The copyright holder for this preprint (which was not certified by peer review) is the author/funder, who has granted bioRxiv a license to display the preprint in perpetuity. It is made available under aCC-BY 4.0 International license.

Figure S1

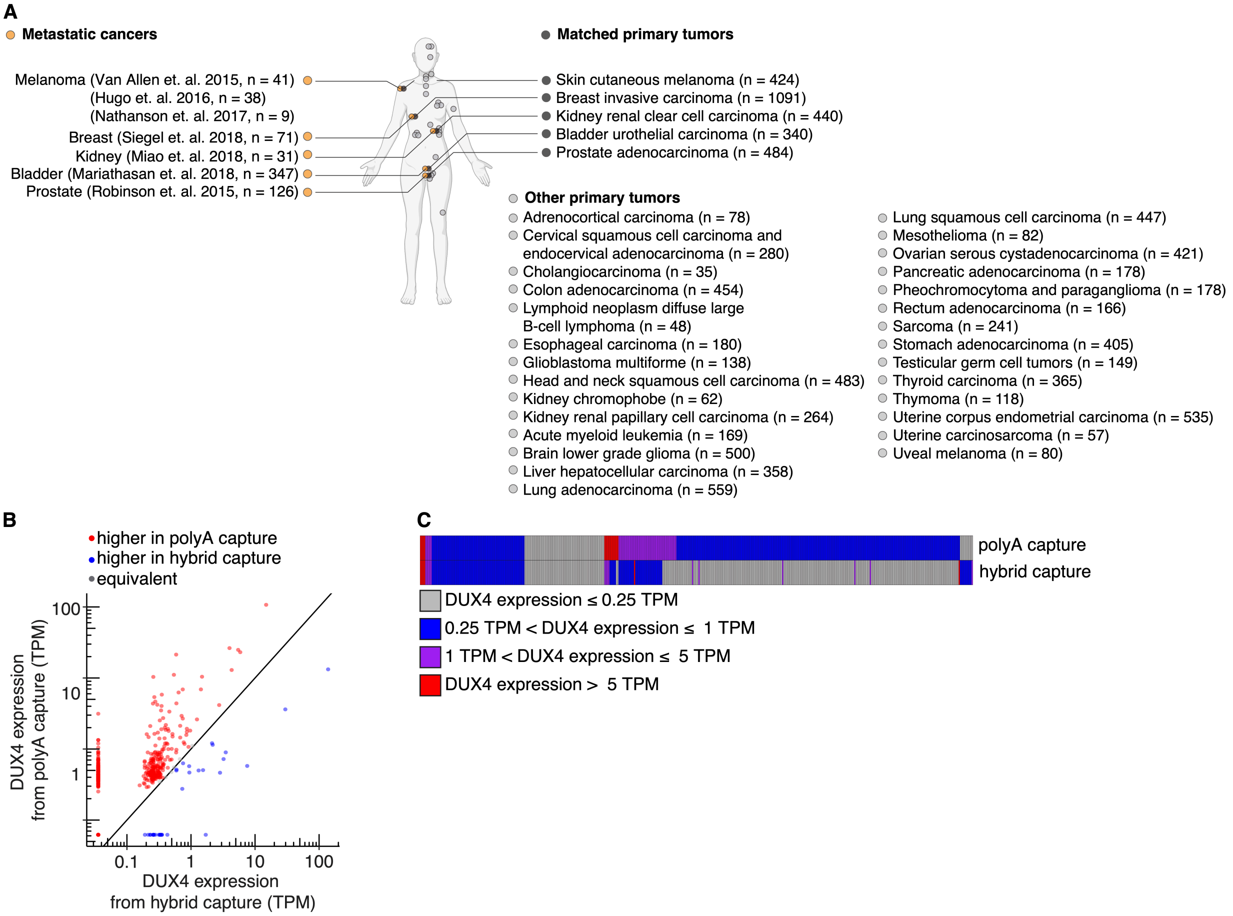

Figure S1. The *DUX4* transcript is likely poly-adenylated.

(**A**) As in (Figure 1A), but the primary cancer cohorts without matched normal sample analyzed in our study are shown.

(**B**) A comparison of *DUX4* expression values (TPM, transcripts per million) measured from sequencing libraries prepared via polyA capture or hybrid capture.

(**C**) As in (B), but a heatmap where patient samples (columns) were stratified according to the indicated categories of *DUX4* expression.

bioRxiv preprint doi: https://doi.org/10.1101/2023.07.10.548412; this version posted July 11, 2023. The copyright holder for this preprint (which was not certified by peer review) is the author/funder, who has granted bioRxiv a license to display the preprint in perpetuity. It is made available under aCC-BY 4.0 International license.

Figure S2

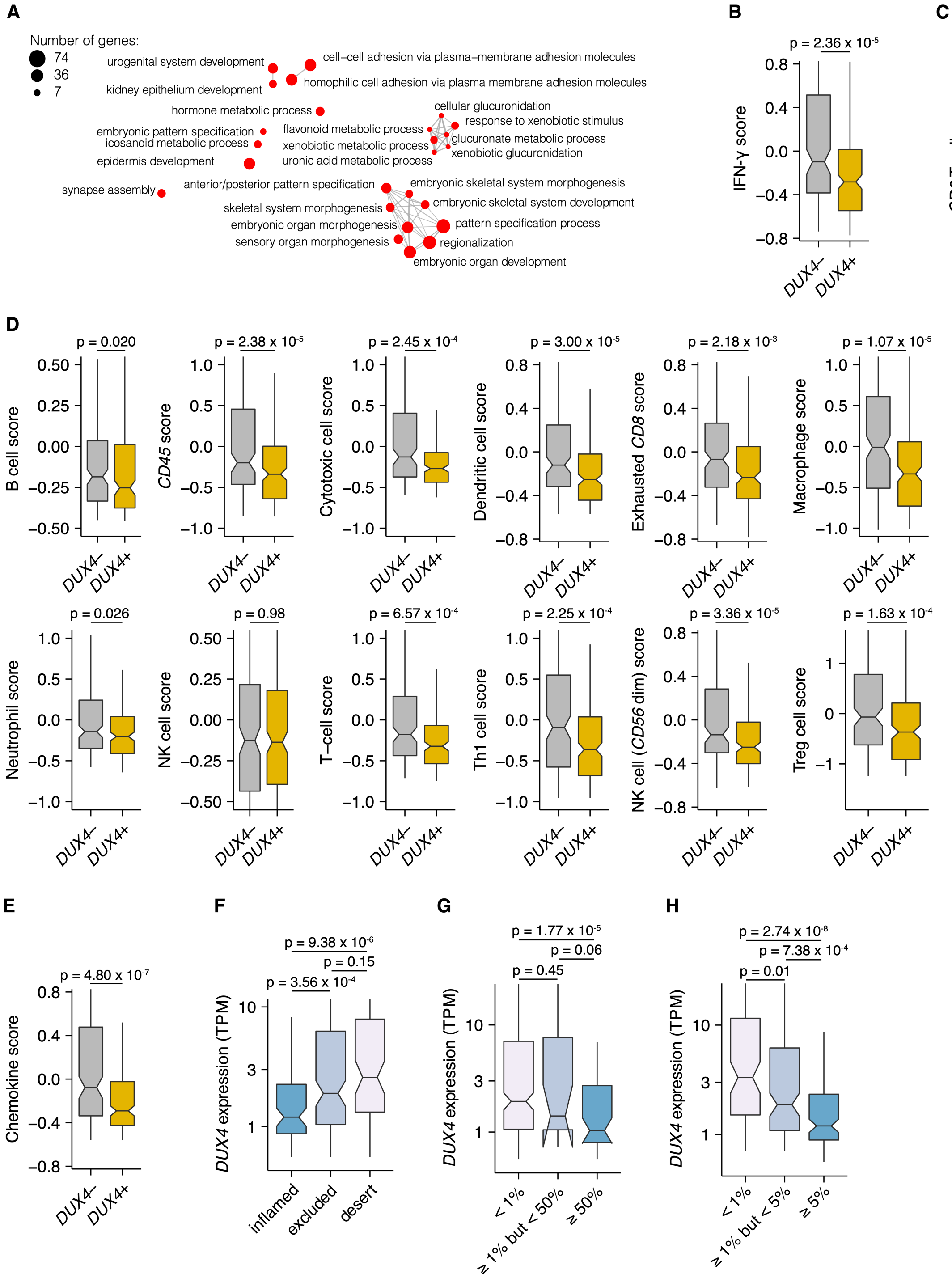

Figure S2. *DUX4*-positivity is correlated with an embryonic gene expression signature, downregulation of interferon-gamma signaling, and exclusion of diverse immune cell types. (**A**) As in (Figure 2A), but the Gene Ontology (GO) enrichment network analysis corresponding to *DUX4*-upregulated genes, compared against the set of coding genes, is shown.

(**B**) Interferon-gamma (IFN-γ) signature score (Ayers et al., 2017). The *p*-value was estimated via a Mann-Whitney *U* test.

bioRxiv preprint doi: https://doi.org/10.1101/2023.07.10.548412; this version posted July 11, 2023. The copyright holder for this preprint (which was not certified by peer review) is the author/funder, who has granted bioRxiv a license to display the preprint in perpetuity. It is made available under aCC-BY 4.0 International license.

(**C**) CD8 T cell score from (Danaher et al., 2017). The *p*-value was estimated via a Mann-Whitney *U* test.
(**D**)As in (**C**), showing the other immune cell signatures available in (Danaher et al., 2017).
(**E**) Chemokine signature score (Coppola et al., 2011). The *p*-value was estimated via a Mann-Whitney *U* test.
(**F**) *DUX4* expression (TPM) in inflamed, immune excluded, and immune desert tumors. The phenotypes are based on $CD8^+$ T cell abundance and degree of tumor infiltration determined by anti-CD8 staining of tumor FFPE sections in the original study (Mariathasan et al., 2018). The *p*-values were estimated via the Mann-Whitney *U* test.
(**G**) *DUX4* expression (TPM) in advanced urothelial carcinoma tumors. The percentage of tumor cells with positive PD-L1 staining are indicated on the x-axis. The *p*-values were estimated via the Mann-Whitney *U* test.
(**H**) As in (**G**), but showing the percentage of tumor-infiltrating immune cells (lymphocytes, macrophages, and dendritic cells) with positive PD-L1 staining on the x-axis.

bioRxiv preprint doi: https://doi.org/10.1101/2023.07.10.548412; this version posted July 11, 2023. The copyright holder for this preprint (which was not certified by peer review) is the author/funder, who has granted bioRxiv a license to display the preprint in perpetuity. It is made available under aCC-BY 4.0 International license.

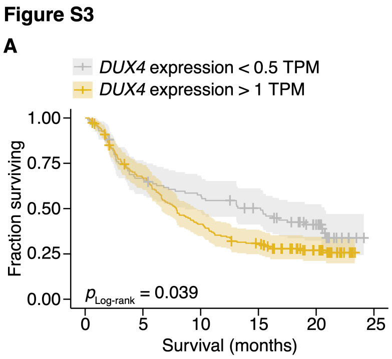

Figure S3. ***DUX4*** **expression status stratifies patients according to survival.**
(**A**) Kaplan-Meier (KM) estimates of overall survival (solid lines), 95% confidence intervals (transparent ribbons), and censored events (crosses) for ICI-treated advanced urothelial carcinoma patients stratified by *DUX4* expression status. The *p*-value was estimated via a log-rank test.

bioRxiv preprint doi: https://doi.org/10.1101/2023.07.10.548412; this version posted July 11, 2023. The copyright holder for this preprint (which was not certified by peer review) is the author/funder, who has granted bioRxiv a license to display the preprint in perpetuity. It is made available under aCC-BY 4.0 International license.

Figure S4

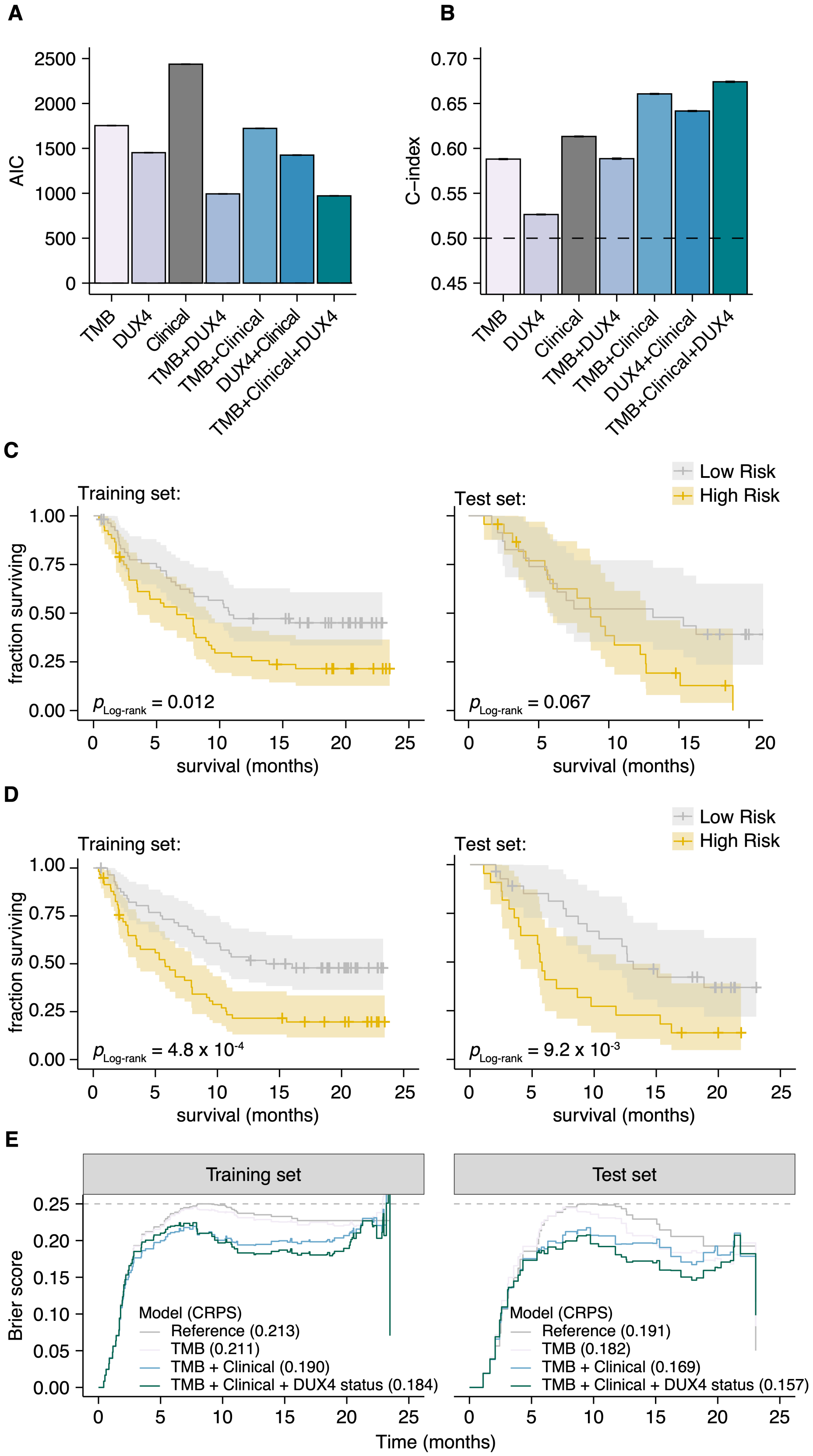

bioRxiv preprint doi: https://doi.org/10.1101/2023.07.10.548412; this version posted July 11, 2023. The copyright holder for this preprint (which was not certified by peer review) is the author/funder, who has granted bioRxiv a license to display the preprint in perpetuity. It is made available under aCC-BY 4.0 International license.

Figure S4. Cox Proportional Hazards regression models containing *DUX4* expression status as a predictor have a better fit to the data.

(**A**) Akaike information criterion (AIC) measurements for goodness of fit for the full (TMB, Clinical, DUX4 expression) vs. reduced Cox PH models, where lower values indicate better fit. The bootstrapped AIC mean and the 95% confidence interval are illustrated. Clinical (ECOG Performance Status and Platinum treatment history).

(**B**) Harrell's concordance indices (C-index) for the full (TMB, Clinical, DUX4 expression) vs. reduced Cox PH models, where high values indicate better model performance. The bootstrapped C-index mean and the 95% confidence interval are illustrated.

(**C**) Kaplan-Meier (KM) estimates of overall survival, 95% confidence interval (transparent ribbon), and censored events (crosses) for low-risk (solid gray line) and high-risk (solid orange line) patients in the training (left) and test (right) sets. Risk group assignments were based on risk scores estimated by the Cox PH model with only TMB as a predictor. *p*-values were estimated via a log-rank test.

(**D**) As in (C), but the risk group assignments were based on risk scores estimated by the Cox PH model with TMB, ECOG Performance Status, and Platinum treatment history as predictors.

(**E)** Time-dependent Brier scores for the full and reduced Cox PH models applied on the training (left) and test (right) sets. The Continuous Ranked Probability Scores (CRPS), defined as the integrated Brier score divided by time, are shown in parentheses. Reference refers to the Kaplan-Meier prediction model. A Brier score = 0.25 indicates random guessing (gray dashed line).

bioRxiv preprint doi: https://doi.org/10.1101/2023.07.10.548412; this version posted July 11, 2023. The copyright holder for this preprint (which was not certified by peer review) is the author/funder, who has granted bioRxiv a license to display the preprint in perpetuity. It is made available under aCC-BY 4.0 International license.

Figure S5

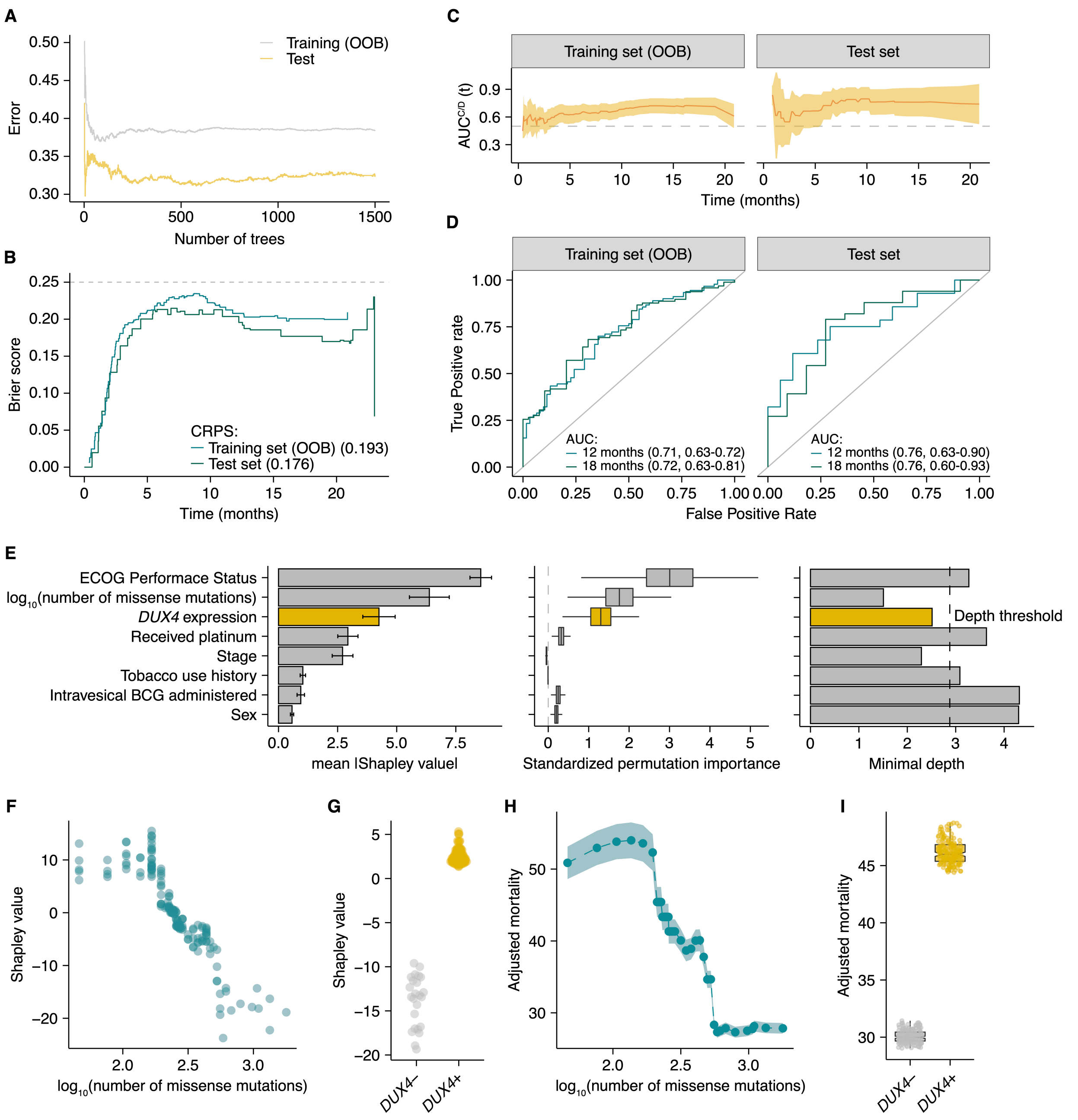

Figure S5. A Random Survival Forest model quantifies the effect of *DUX4* status on overall survival probability in the context of immune checkpoint inhibition.

(**A**) Error (1 – Harrell's concordance index) as a function of the number of trees in the Random Survival Forest (RSF) model. The training out-of-bag error (OOB error, solid gray line) and the test error (solid orange line) are shown. 1500 trees were used in the final model.

(**B**) Time-dependent Brier scores for the RSF model estimated from the training (solid turquoise line) or test (solid teal line) sets. OOB survival predictions were used to calculate the Brier score for the training set. The Continuous Ranked Probability Scores (CRPS), defined as the integrated Brier score divided by time, for both sets are shown. A Brier score = 0.25 indicates random guessing (gray dashed line).

bioRxiv preprint doi: https://doi.org/10.1101/2023.07.10.548412; this version posted July 11, 2023. The copyright holder for this preprint (which was not certified by peer review) is the author/funder, who has granted bioRxiv a license to display the preprint in perpetuity. It is made available under aCC-BY 4.0 International license.

(**C**) Time-dependent ROC analyses. The $AUC^{C/D}$ (solid orange line) and the 95% confidence interval (transparent orange ribbon) are shown over the observation time for the training and test sets. The OOB mortality predictions were used to calculate the training set $AUC^{C/D}$.
(**D**) The Receiver Operating Characteristic (ROC) curves for the RSF model at 12 (solid turquoise line) and 18 (solid teal line) months. The Cumulative/Dynamic Area Under the ROC Curve ($AUC^{C/D}$) and 95% confidence interval are specified. The OOB mortality predictions were used to calculate the training set $AUC^{C/D}$.
(**E**) RSF feature importance. The mean absolute Shapley values and the 95% confidence intervals are shown (left). The standardized permutation importance and confidence regions estimated via delete-*d* jackknife subsampling are plotted (middle); noise variables are indicated by permutation importance measures ≤ 0. The minimal depth measures are shown (right); variables with values exceeding the depth threshold (gray dashed line) are designated as noise variables.
(**F**) Shapley dependence plot illustrating the relationship between tumor mutational burden (TMB, number of missense mutations) and mortality. Each point corresponds to a single patient.
(**G**) As in (**F**), but showing *DUX4* expression status.
(**H**) Partial plot illustrating the marginal effect of TMB on mortality. Each point corresponds to the average RSF mortality predictions when TMB is fixed to the indicated value for all patients. The transparent ribbon corresponds to the 95% confidence interval.
(**I**) Partial plot illustrating the marginal effect of *DUX4* expression status. The points correspond to the RSF prediction for mortality for each patient when *DUX4* expression status is fixed to the indicated value for the entire cohort.

bioRxiv preprint doi: https://doi.org/10.1101/2023.07.10.548412; this version posted July 11, 2023. The copyright holder for this preprint (which was not certified by peer review) is the author/funder, who has granted bioRxiv a license to display the preprint in perpetuity. It is made available under aCC-BY 4.0 International license.

Table S1. Cox Proportional Hazards Regression for Overall Survival (TGFB1 expression included)

	Univariate				Multivariate[a,b]			
Characteristic	HR	95% CI	p-value	q-value[c]	HR	95% CI	p-value	q-value[c]
Tumor mutational burden[d]	0.43	0.28 - 0.66	1.10×10^{-4}	8.27×10^{-4}	0.14	0.06 - 0.30	8.41×10^{-7}	1.26×10^{-5}
DUX4 expression:								
< 0.25 TPM (reference)	—	—	—	—	—	—	—	—
> 1 TPM	1.85	1.10 - 3.11	0.021	0.079	3.12	1.52 - 6.38	1.87×10^{-3}	7.01×10^{-3}
ECOG Performace Statuts[e]:								
0 (reference)	—	—	—	—	—	—	—	—
1	2.10	1.58 - 2.79	2.95×10^{-7}	4.43×10^{-6}	2.86	1.79 - 4.57	1.18×10^{-5}	8.84×10^{-5}
2	1.97	1.04 - 3.73	0.036	0.091	5.31	1.79 - 15.7	2.58×10^{-3}	7.73×10^{-3}
Received platinum:								
No (reference)	—	—	—	—	—	—	—	—
Yes	1.41	1.01 - 1.98	0.047	0.10	2.69	1.47 - 4.93	1.32×10^{-3}	6.61×10^{-3}
Intravesical BCG administered:								
No (reference)	—	—	—	—	—	—	—	—
Yes	0.98	0.73 - 1.32	0.89	0.92	0.64	0.38 - 1.06	0.08	0.15
Tobacco use history:								
Never (reference)	—	—	—	—	—	—	—	—
Previous	0.90	0.68 - 1.19	0.45	0.61	1.21	0.78 - 1.87	0.39	0.45
Current	1.02	0.65 - 1.61	0.92	0.92	1.43	0.76 - 2.67	0.27	0.36
Stage:								
I	—	—	—	—	—	—	—	—
II	0.96	0.69 - 1.35	0.83	0.92	1.61	0.91 - 2.86	0.10	0.17
III	1.23	0.86 - 1.75	0.25	0.38	1.62	0.96 - 2.74	0.07	0.15
IV	0.98	0.68 - 1.42	0.92	0.92	0.46	0.24 - 0.88	0.020	0.050
Sex:								
Male (reference)	—	—	—	—	—	—	—	—
Female	1.23	0.91 - 1.66	0.18	0.31	0.76	0.47 - 1.23	0.26	0.36
TGFB1 expression:								
≤ 25th percentile (Q1, reference)	—	—	—	—	—	—	—	—
> 25th percentile and ≤ 50th percentile (Q2)	1.30	0.88 - 1.91	0.19	0.31	0.75	0.40 - 1.41	0.38	0.45
> 50th percentile and ≤ 75th percentile (Q3)	1.52	1.04 - 2.23	0.031	0.091	1.04	0.55 - 1.95	0.90	0.91
> 75th percentile (Q4)	1.68	1.16 - 2.45	6.46×10^{-3}	0.032	0.96	0.50 - 1.87	0.91	0.91

[a]Log-rank test: p-value = 1.27×10^{-7}
[b]Akaike Information Criterion = 975.30; Bayesian Information Criterion = 1015.67; Harrell's Concordance Index = 0.71
[c]Benjamini-Hochberg FDR correction
[d]$\log_{10}$(number of missense mutations)
[e]Eastern Oncology Cooperative Group Performance Status
